# Comparative Characterization of the Complete Mitochondrial Genomes of the Three Apple Snails (Gastropoda: Ampullariidae) and the Phylogenetic Analyses

**DOI:** 10.3390/ijms19113646

**Published:** 2018-11-19

**Authors:** Huirong Yang, Jia-en Zhang, Jun Xia, Jinzeng Yang, Jing Guo, Zhixin Deng, Mingzhu Luo

**Affiliations:** 1 College of Marine Sciences, South China Agricultural University, Guangzhou 510640, China; hry@scau.edu.cn; 2 Department of Human Nutrition, Food and Animal Sciences, University of Hawaii at Manoa, Honolulu, HI 96822, USA; xiajun2004263@163.com (J.X.); jinzeng@hawaii.edu (J.X.); 3 Institute of Tropical and Subtropical Ecology, South China Agricultural University, Guangzhou 510642, China; jingaj@163.com (J.G.); zhxdeng@scau.edu.cn (Z.D.); lmzhd2701@scau.edu.cn (M.L.); 4 Xinjiang Acadamy of Animal Sciences, Institute of Veterinary Medicine (Research Center of Animal Clinical), Urumqi 830000, China; 5 Guangdong Engineering Research Center for Modern Eco-Agriculture and Circular Agriculture, Guangzhou 510642, China

**Keywords:** *Pomacea canaliculata*, *Pomacea diffusa*, *Pomacea maculate*, Ampullariidae, mitochondrial genome, comparative characterization, phylogenetic analysis

## Abstract

The apple snails *Pomacea canaliculata*, *Pomacea diffusa* and *Pomacea maculate* (Gastropoda: Caenogastropoda: Ampullariidae) are invasive pests causing massive economic losses and ecological damage. We sequenced and characterized the complete mitochondrial genomes of these snails to conduct phylogenetic analyses based on comparisons with the mitochondrial protein coding sequences of 47 Caenogastropoda species. The gene arrangements, distribution and content were canonically identical and consistent with typical Mollusca except for the *tRNA-Gln* absent in *P. diffusa*. An identifiable control region (d-loop) was absent. Bayesian phylogenetic analysis indicated that all the Ampullariidae species clustered on the same branch. The genus *Pomacea* clustered together and then with the genus *Marisa*. The orders Architaenioglossa and Sorbeoconcha clustered together and then with the order Hypsogastropoda. Furthermore, the intergenic and interspecific taxonomic positions were defined. Unexpectedly, *Ceraesignum maximum*, *Dendropoma gregarium*, *Eualetes tulipa* and *Thylacodes squamigerus*, traditionally classified in order Hypsogastropoda, were isolated from the order Hypsogastropoda in the most external branch of the Bayesian inference tree. The divergence times of the Caenogastropoda indicated that their evolutionary process covered four geological epochs that included the Quaternary, Neogene, Paleogene and Cretaceous periods. This study will facilitate further investigation of species identification to aid in the implementation of effective management and control strategies of these invasive species.

## 1. Introduction

Apple snails (Ampullariidae) are freshwater snails distributed naturally throughout the humid tropics and subtropics and *Pomacea* is the largest of nine genera in the family. *Pomacea* spp. are indigenous to South America and were introduced from Argentina to Taiwan for commercial purposes. They have subsequently appeared in numerous countries throughout southern and eastern Asia including China. However, these animals have become pests of wetland rice and other crops causing massive economic losses. They have spread into nonagricultural wetlands and their ecological impacts in these habitats are more difficult to estimate [1,2].

*P. canaliculata* is listed among the world’s 100 worst invasive species by the United States Aquatic Nuisance Species Task Force (USANSTF) [3]. However, the identification and taxonomy of the genus *Pomacea* are difficult and confusing due to the highly conserved external morphology across the genus despite considerable intraspecific shell variation. This has obscured the true number of species and their identities [4,5,6].

Animal mitochondrial DNAs (mtDNA) are 16–19 kb circular molecules comprised of 37 genes encoding 22 transfer RNAs (tRNA), 13 proteins, two ribosomal RNAs and a control region (CR) that possesses cis regulatory elements [7,8]. The compactness, maternal inheritance, rapid evolutionary rate and short coalescence time compared to nuclear DNA have resulted in the use of mtDNA sequence information for phylogenic studies, taxonomic resolution [9] and population genetics [10].

Currently, *P. canaliculata*, *Pomacea maculate* (formerly *Pomacea insularum*) and *Pomacea diffusa* are the most widespread species in China. Most studies of the genus *Pomacea* actually refer to *P. canaliculata.* This species has been defined in terms of its life history, reproduction and as an intermediate host for the rat lungworm *Angiostrongylus cantonensis*. Control and management strategies have been largely based on this species [11,12,13,14]. In contrast, there are few studies of *P. maculata* and *P. diffusa* [15,16,17,18,19,20].

The Caenogastropoda is the most diverse clade of the Gastropoda and their evolution and population biology are under active investigation. However, phylogenetic analyses based on mtDNA genome have not been completely investigated and only preliminary and limited mtDNA information is available for the apple snails [20,21,22,23,24]. The complete mtDNA genome sequences of the family Ampullariidae are currently under investigation [20,23,25].

We sequenced complete mt genomes of the three apple snails *P. canaliculata*, *P. maculata* and *P. diffusa* using next-generation sequencing (NGS) technology to analyze their phylogeny and evolutionary histories. The identification of new DNA markers and taxonomic viewpoints may facilitate further species identification and assist the development of more effective management and control strategies of these invasive species.

## 2. Results and Discussion

### 2.1. Genome Organization and Composition

**Figure 1:**
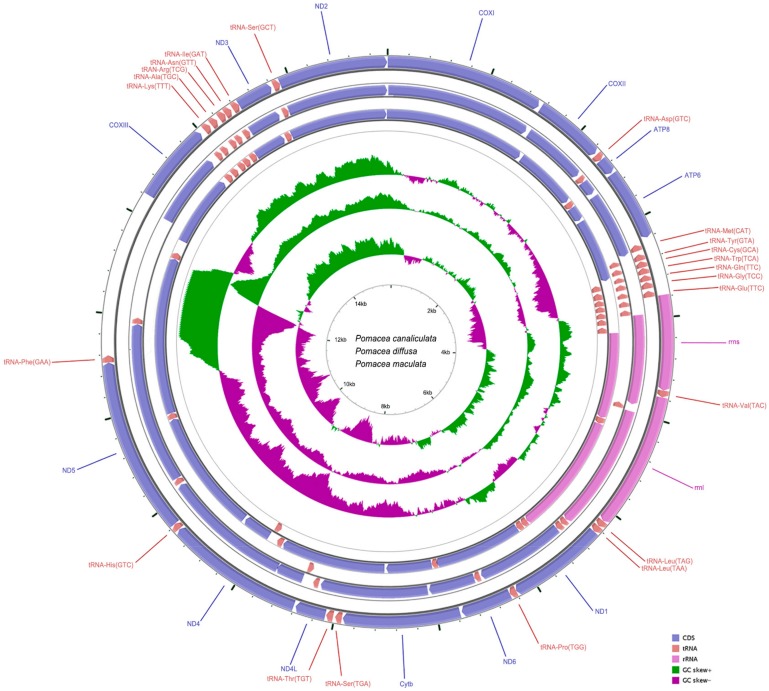
Gene map of the complete mt genomes for *P. canaliculata* (GenBank accession No. KU052865), *P. diffusa* (KY008698) and *P. maculate* (KY008699). The larger ring indicates gene arrangement and distribution. The smaller ring indicates the GC content. *ND1-6*: NADH dehydrogenase subunits 1–6; *COXI-III*: cytochrome c oxidase subunits 1–3; *ATP6* and *ATP8*: ATPase subunits 6 and 8; *Cytb*: cytochrome b; *rrn*: ribosomal RNA genes; *tRNA*: transfer RNA gene.

The complete mt genomes of *P. canaliculata*, *P. diffusa* and *P. maculate* were 16,965, 16,640 and 15,516 bp in length. These contained the canonical 13 PCGs (Protein Coding Genes), two rRNA genes (*rrnl* and *rrns*) and 22 *tRNA* genes with the exception of *tRNA-Gln* that was absent in *P. diffusa*. Most of the 37 genes are encoded on the heavy strand (H-strand) as in most Mollusca. However, seven *tRNA* genes (*Met*, *Tyr*, *Cys*, *Trp*, *Gln*, *Gly* and *Glu*) from all three species as well as *tRNA-Val* of *P. diffusa* and *tRNA-Thr* of *P. diffusa* and *P. maculate* are present on the light strand (L-strand). The G + C content varied from 14.71% to 41.18% (Table 1). The large and small subunit rRNA genes *rrnl* and *rrns* were located between *tRNA-Leu* (TAG) and *tRNA-Glu* (TTG) genes and separated by the *tRNA-Val* (TCG) gene (Figure 1). The absence of a specific and identifiable control region is consistent with other Gastropoda [26,27,28,29].

Gene arrangement and distribution of the three apple snails were canonically identical and consistent with typical Mollusca [30,31,32,33,34,35] but obviously different from vertebrates. We identified the presence of 24, 26 and 24 intergenic spacers (IGS) for *P. canaliculata*; *P. diffusa* and *P. maculate* totaling 2020, 1684 and 547 bp, respectively. The largest IGS was located between *tRNA-Phe*―*COXIII* IGS on the H strand and was 1617 bp in *P. canaliculata*, 1073 bp in *P. diffusa* and 141 bp in *P. maculate*. These regions accounted for the shorter mitogenome in *P. maculate* (Table 2). MtDNA-IGS are regions of non-coding DNA between genes as well as between the tandem rRNA gene copies. The mtDNA genes in animals generally have very short spacers and are important for evolutionary studies because they change more rapidly than gene sequences. IGS regions are thought to have sequence-independent functions [36]. Additionally, a 119 bp IGS between *tRNA-Trp* and *tRNA-Gly* in *P. diffusa* accounted for the absence of the *tRNA-Gln*.

Mitochondrial genes overlap and serve to compact the mitogenomes. Each of the snails possessed five overlap sites totaling 11 bp in *P. canaliculata*, 27 bp in *P. diffusa* and 13 bp in *P. maculate* similar to other mitogenomes [37,38,39,40,41,42,43,44,45].

The gene overlaps we identified were present in *ND1-tRNA-Pro* of *P. canaliculata*, *ND4L-ND4* and *ND5-tRNA-Phe* of *P. diffusa*, *tRNA-Leu-tRNA-Leu* and *ND1-tRNA-Pro* of *P. maculate.* (Table 2). Generally, we allowed gene overlaps between adjacent genes but not between protein coding genes and *tRNAs*. When a full termination codon (UAA or UAG) caused an overlap between a protein-coding gene and a *tRNA*, we preferred to annotating this gene with incomplete termination codon U/UA rather than an overlap. The incomplete termination codons were not adopted for the three apple snails. The annotation principles were determined by the annotated species available on the website data, which had close relationships with them.

### 2.2. Nucleotide Composition

The overall nucleotide compositions of the H-strand of the three apple snails in descending order were 40.20% T, 31.18% A, 15.59% G and 13.03% C, with an average G + C content of 28.62% indicating significant strand asymmetry or strand-specific bias commonly observed in the Mollusca [31,35,38,40]. Using the GC-/AT skew analysis, we found that the H-strand showed an over representation of T and A as compared to the L-strand. Strand asymmetry in the nucleotide composition was also reflected in the codon usage of genes oriented in opposite directions. The genes encoded on the H-strand showed clear preference for T or A to end codons, whereas G or C ending codons were over represented on the L-strand. The underlying mechanism responsible for the strand bias has been generally interpreted as evidence of an asymmetrical directional mutation pressure. This is associated with replication processes when one strand remains transiently in a single-stranded state, making it more vulnerable to DNA damage [46,47].

The highest A + T content of the genomes was detected in *tRNA-Asp* and was 85.29% in *P. canaliculata*, 82.35% in *P. diffusa*, and 83.82% in *P. maculate*. The highest G + C contents were 41.18% in the *tRNA-Tyr* of *P. diffusa*, 36.15% in the *COXIII* of *P. canaliculata*, and 35.29% in the *tRNA-Thr* of *P. maculate*. These phenomena were consistent with the findings of previous reports on other Mollusca [30,32,39,48].

### 2.3. Protein Coding Genes (PCG)

**Figure 2:**
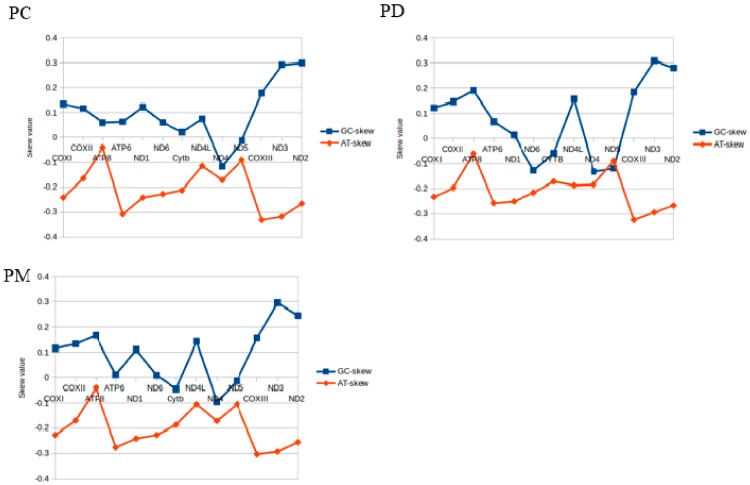
Graphical illustration showing the AT- and GC-skew in the PCGs of the mitochondrial genome of *P. canaliculata* (**PC**), *P. diffusa* (**PD**) and *P. maculate* (**PM**).

The 13 PCGs were represented in the apple snail mitogenomes and were H-strand encoded. These included three subunits of cytochrome c oxidase (*COXI-III*), seven subunits of NADH ubiquinone oxidoreductase complex (*ND1-6*, *ND4L*), a cytochrome b oxidoreductase complex (*Cyt b*) and two subunits of ATP synthases (*ATP6* and *ATP8*). The latter ranged in size from 159 bp (*ATP8*) to 1728 bp (*ND5*) comprising 11,220–11,268 bp total accounting for 66.14–72.62% of the entire mitogenomes. Each PCG length was identical between the three snails with the exception of *ND4L*, *ATP6*, *ND1*, *ND2*, *ND4* and *ND5* that differed by 6–21 bp. The AT-skew of the 13 PCGs among the three species were all negative and the majority of the GC skew values were positive. The AT skews of *COXIII* and *ND3* were the lowest and the GC skews of *ND2* and *ND3* were the highest (Figure 2).

We identified initiation and termination codons based on alignments with the corresponding genes of other snails. The mitogenomes of the apple snails exhibited a canonical genetic code shared by all Mollusca. An orthodox initiation codon AUG was used for most of the protein-coding genes. In contrast, AUA was used in *ND4L*, *ND4* and *COXIII* of *P. maculate* and in *ND4* and *COXIII* of *P. canaliculata*. These were also the accepted canonical mitochondrial initiation codons for Mollusca mt genomes (Table 2). Termination codons were represented by only UAA and UAG for all three apple snails and UAA was the most abundant. We did not identify any incomplete termination codons although they are common in other Gastropoda [49,50].

**Figure 3:**
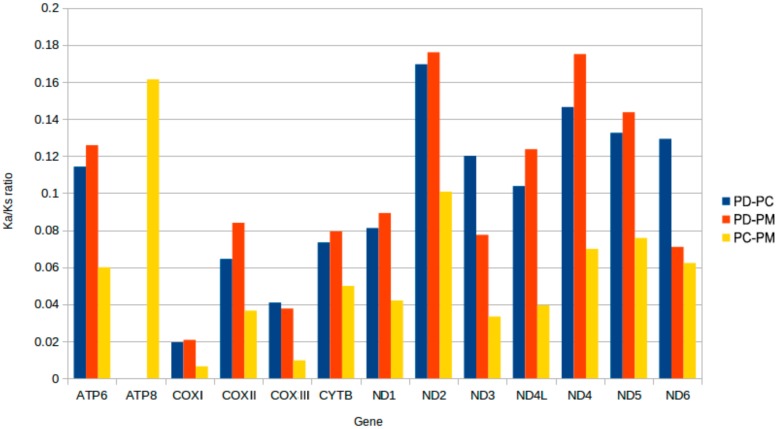
Evolutionary rates of the mitochondrial genome of *P. canaliculata* (**PC**), *P. diffusa* (**PD**) and *P. maculate* (**PM**). The rates of non-synonymous substitutions to the rate of synonymous substitutions (Ka/Ks) for each PCG are indicated.

The rates of nonsynonymous amino acid substitutions over synonymous silent substitution (Ka/Ks) for the 13 protein-coding genes were 0.0837. These varied from 0.0065 in *COXI* to 0.1761 in *ND2*. This indicated that the functional genes evolved under strong purifying selection, which meant natural selection against deleterious mutations with negative selective coefficients [51] (Figure 3). The selection pressures differed among the genes and they likely evolved in different ways [52]. Interestingly, the *ND2* genes possessed the highest ratios indicating that the selection pressures were strand independent.

**Figure 4:**
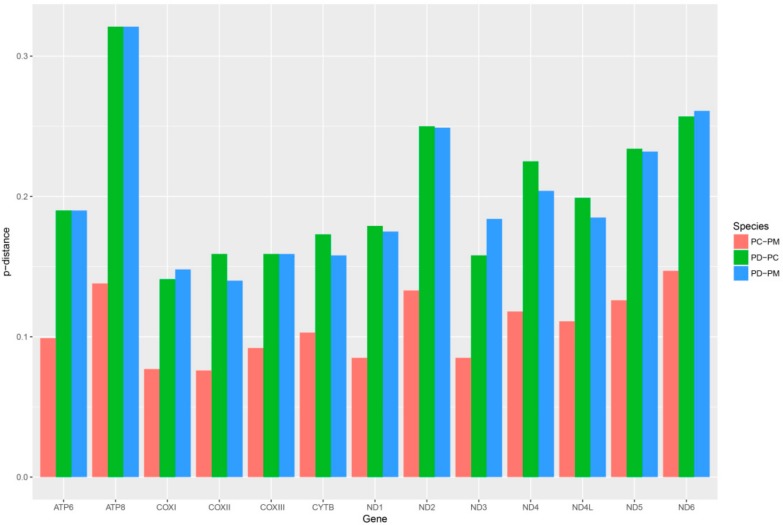
Overall mean p-genetic distances between *P. canaliculata*, *P. diffusa* and *P. maculate* for each of 13 PCG. Values were calculated using the first and second nucleotide positions, the total codon and over the full sequence.

The conservation of mtDNA genes was evaluated based on the overall p-genetic distance among *P. canaliculata* (PC), *P. diffusa* (PD) and *P. maculate* (PM) (Figure 4). Comparisons of full-length PCGs indicated that *COXI* and *COXII* had the lowest overall p-genetic distances (0.122, 0.125) and *ATP8* and *ND6* had the highest (0.260, 0.222). Furthermore, the values based on the first and second nucleotides of codons were also consistent with those based on data of the full-length codons. This indicated that *ATP8* and *ND6* likely had the highest evolutionary rates while *COXI* and *COXII* had the lowest. The p-genetic distances between PD-PC and PD-PM was much more higher than that for PC-PM. This meant that the PCG sequences of *P. canaliculata* and *P. maculate* were the most similar among the three apple snails.

### 2.4. Mitochondrial Gene Codon Usage

**Figure 5:**
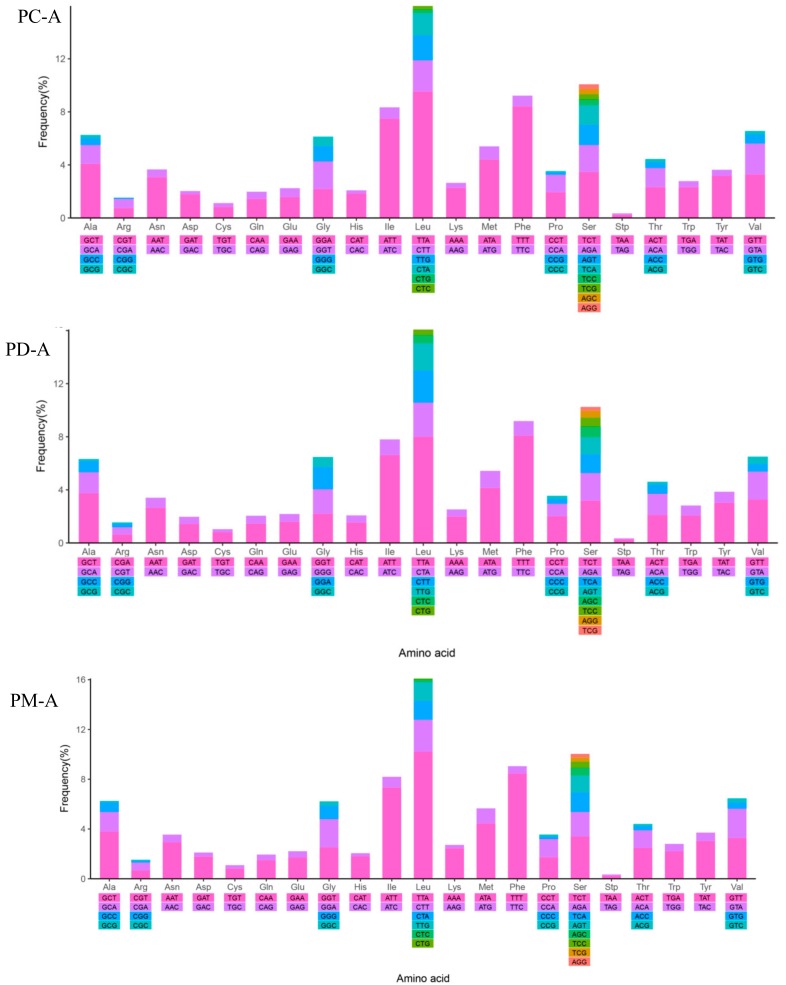

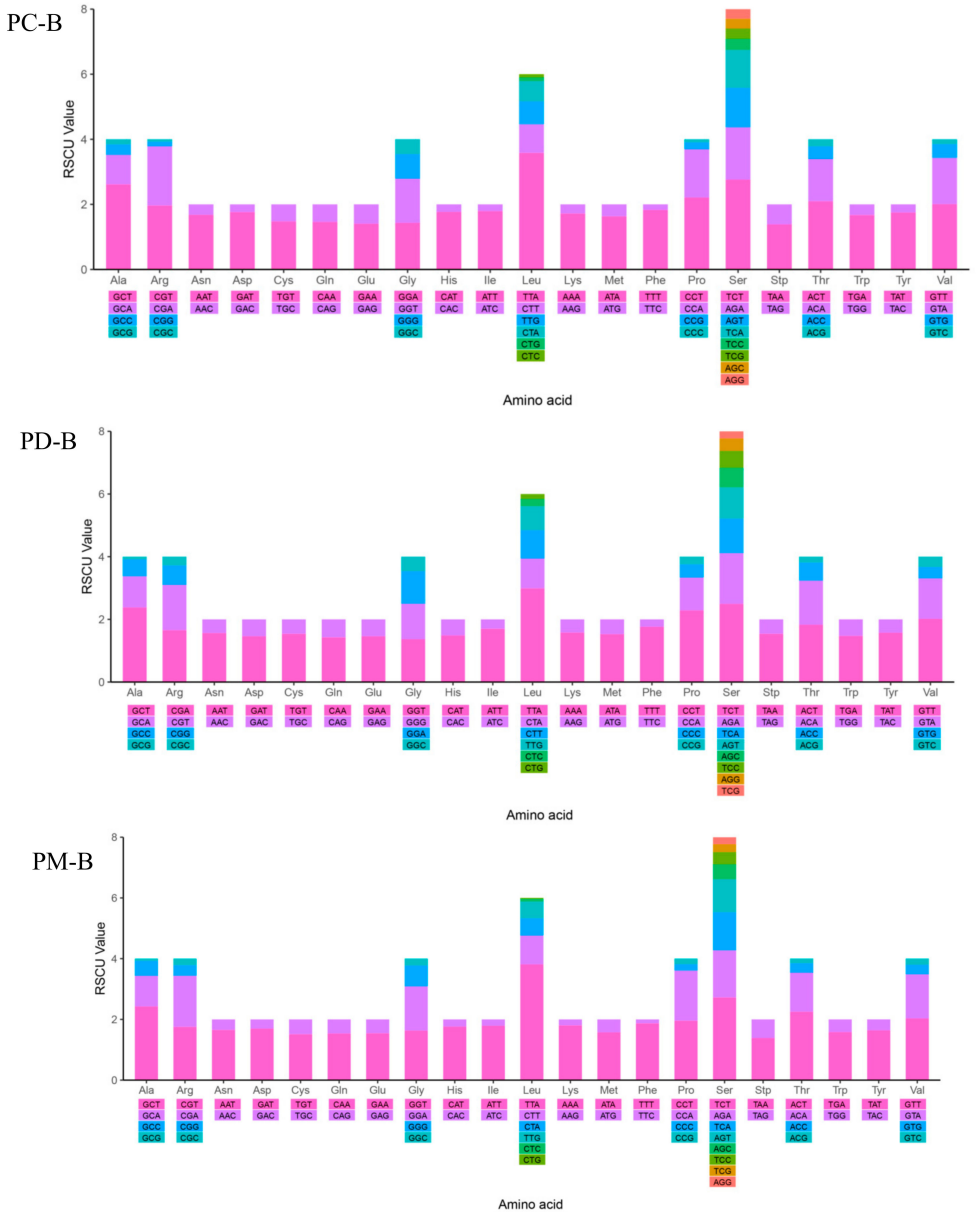
Codon Frequency (**A**) and RSCU (**B**) in the mitochondrial genomes of *P. canaliculata* (**PC**), *P. diffusa* (**PD**) and *P. maculate* (**PM**). PC-A indicated the Codon Frequency of *P. canaliculata*; PD-A indicated the Codon Frequency of *P. diffusa*; PM-A indicated the Codon Frequency of *P. maculate*. PC-B indicated the RSCU of *P. canaliculata*; PD-B indicated the RSCU of *P. diffusa*; PM-B indicated the RSCU of *P. maculate*.

The amino acids Ser and Leu were utilized by eight and six different codons, respectively, while all other amino acids were encoded by either two or four. The frequencies in the PCGs of Phe (UUU), Leu (UUA), Ile (AUU), Met (AUA), and Ala (GCU) were the most frequently used and Stop (UAG) and Arg (CGC) were least frequently used. However, the relative synonymous codon usage (RSCU) analysis indicated that Leu (UUA), Ser (UCU) and Ala (GCU) were the most frequent while Leu (CUC, CUG), Pro (CCC, CCG), Arg (CGC) and Ala (GCG) were rare. Moreover, the GC content and the AT and GC skew of the PCGs were reflected in the codon usage and were closely related and consistent (Table 1; Figure 2) [53,54,55]. The RSCU values indicated that codons with A or U in the third position were more frequent than C or G. Therefore, codons NNA and NNU were in the majority while the synonymous codons NNC and NNG were in the minority (Figure 5).

### 2.5. Transfer and Ribosomal RNA Genes

The mitogenomes of the apple snails each contained 22 *tRNA* genes varying from 62 bp (*tRNA-Gln* and *tRNA-Cys*) to 72 bp (*tRNA-Ile*) and were typical of metazoan mitogenomes. In general, there was a one-to-one correspondence of codon and anticodon. However, serine was determined by two types of anticodons (UGA, GCU) and leucine was determined by UAG and UAA in the apple snails, identical to other Mollusca [30,34]. All 22 *tRNA* genes (*tRNA-Gln* absent in *P. diffusa*) were predicted to be folded into canonical cloverleaf secondary structures as predicted using the *tRNA* Scan-SE software (http://lowelab.ucsc.edu/tRNAscan-SE/) [56], although numerous non-complementary and U-G base pairs existed in the stem regions. Stem mismatches are common for mitochondrial *tRNA* genes and are repaired via post-transcriptional editing [57].

*tRNA* genes were predicted using a Cove score cutoff of 0.1 by tRNA scan-SE v.2.0, combined with ARWEN software (www.acgt.se/online.html) and then confirmed by MitoFish (http://mitofish.aori.u-tokyo.ac.jp/) to confirm the annotation. We found high similarity in the corresponding *tRNA-Gln* sequences among the three snails. In addition, the secondary structure of *tRNA-Gln* in *P. diffusa* could not be predicted, but the *tRNA-Gln* in *P. canaliculata* and *P. maculate* could be done successfully. We are unsure whether this gene segment variation led to the unsuccessful prediction of secondary structure or was deficiency true deficiency of *tRNA-Gln* in *P. diffusa*. The mitochondrial genes of other snails have not been completely investigated and only preliminary and limited mtDNA information is available. Further research is needed to clarify this situation.

We also identified H-strand large (*rrnl*) and small subunit (*rrns*) rRNA genes ranging in size from 929 to 950 bp and 1331 and 1345 bp, respectively. These were positioned between *tRNA-Glu* (UAG) and *tRNA-Leu* (UUC) *tRNA-Val* (UAC) and consistent with most Mollusca [31,33,48].

The AT content of *rrns* ranged from 70.32 and 71.91% with an AT-skew of 0.012–0.0377 and a GC-skew from 0.1773 to 0.249. The AT content of the *rrnl* gene ranged from 72.86 to 74.53% with an AT-skew of 0.0051–0.0163 and a GC-skew from 0.1397 to 0.1622. This nucleotide distribution differed when compared with the entire mitogenome; the protein-coding genes and *tRNAs* all had a T and G bias.

### 2.6. Mitogenome Comparisions

**Figure 6:**
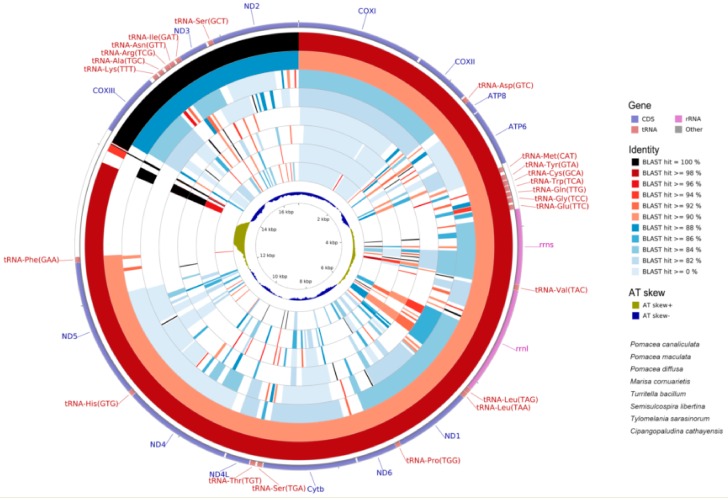
Graphical map of the BLAST results showing nucleotide identity between the complete mitochondrial genomes of *P. canaliculata* and species from the orders Sorbeoconcha and Architaenioglossa. BLAST results are presented in order with the sequence most similar to the reference (*P. canaliculata*) placed closer to the outer edge of the map. The gene regions, Blast identities and AT skew are depicted from outside to inside on the circle. The species from outside to inside are, respectively, *P. canaliculata* (KU052865), *P. maculata* (KY008699), *P. diffusa* (KY008698), *M. cornuarietis* (NC_025334), *T. bacillum* (NC_029717), *S. libertine* (NC_023364), *T. sarasinorum* (NC_030263) and *C. cathayensis* (NC_025577).

**Figure 7:**
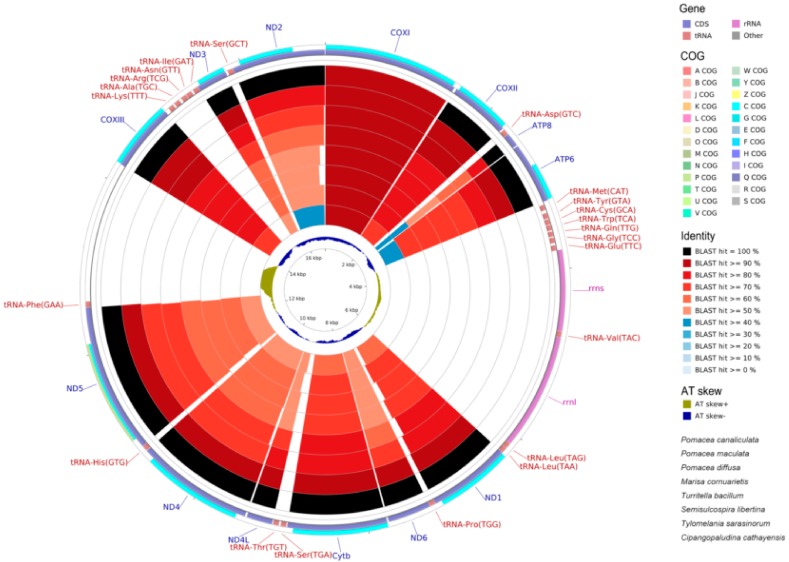
Graphical map of the BLAST results of mitochondrial CDS identities between *P. canaliculata* (NC_024586) and Sorbeoconcha and Architaenioglossa species. The sequences most similar to the reference (*P. canaliculata*) are placed closer to the outer edge of the map. COG (Clusters of Orthologous Groups of proteins), gene region, Blast identity and AT skew are shown from outside to inside. The species from outside to inside are named as in Figure 6.

**Figure 8:**
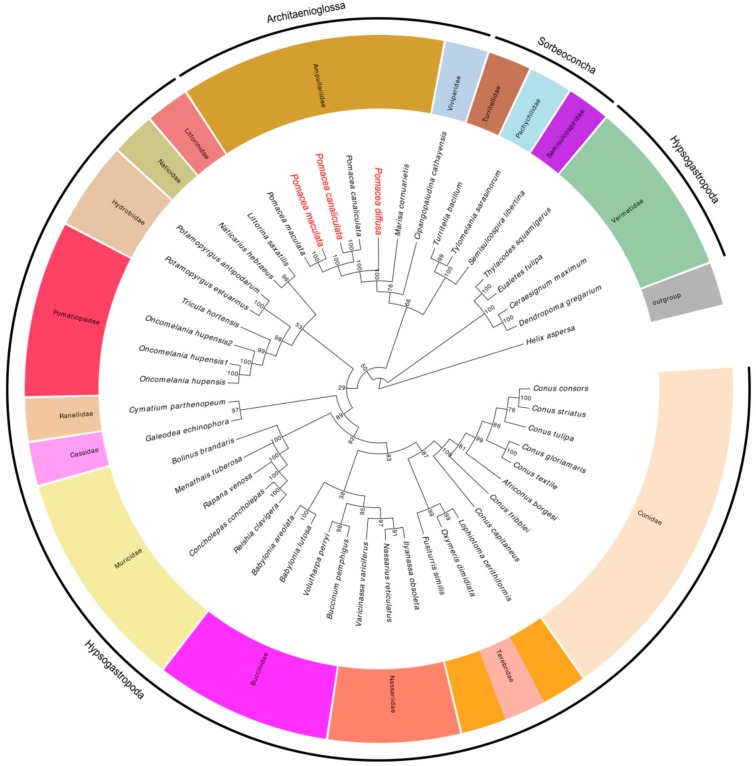
Bayesian inference tree inferred from the amino acid sequences of 13 PCGs of 47 species of Caenogastropoda. *Helix aspersa*, NC_021747, was used as the outgroup. The numbers at the nodes represent the Bayesian posterior probabilities. Species in red indicate sequences generated in this study.

We compared the mitochondrial genomes of *P. canaliculata* with other available mitogenomes in the orders Sorbeoconcha and Architaenioglossa of the Caenogastropoda. Both the nucleotide (Figure 6) and CDS (coding DNA sequence) (Figure 7) regions were highly similar. *ATP8* was the least conserved over in all species with a 59.34% similarity that was appropriate for the phylogenetic and evolutional analyses within the family Ampullariidae or genus Pomacea. On the contrary, *COXI* was the most conservative with the highest similarity (95.41%) and appropriate for the analyses above the family Ampullariidae (Figure 7). The average similarities for the 13 CDS genes were 93.96% (*P. maculate*), 82.41% (*P. diffusa*), 77.62% (*M. cornuarietis*), 68.92% (*Semisulcospira libertine*), 68.24% (*Turritella bacillum*) and 67.39% (*Tylomelania sarasinorum*). *P. canaliculata* was most closely related to *P. maculate* and then with *P. diffusa* and *M. cornuarietis* (Figure 8). Since nucleotide variation and diversity is greater than that for CDS regions, the mitogenome nucleotides showed less identity. This difference was also consistent when the wobble nucleotides are considered, so it was a more effective tool for phylogenetics (compare Figure 6 and Figure 7).

### 2.7. Phylogenetic Analyses

Phylogenetic analyses were carried out based on the concatenated alignment of amino acid sequences of 13 PCGs covering three orders, 18 families, 32 genera and 47 species of Caenogastropoda. We selected *Helix aspersa* (NC_021747) (Gastropoda, Heterobranchia) as the out-group (Table S1). Bayesian and Maximum Likelihood (ML) analyses produced almost identical topologies with similar branch lengths and strong bootstraps (ML) and posterior probabilities (Bayesian) values (Figure 8). We preferred the phylogenetic analyses based on the PCG information because the 47 Caenogastropoda species belonged to different orders i.e., Architaenioglossa, Hypsogastropoda and Sorbeoconcha and possessed wide-ranging taxonomies. Multiple amino acid sequence alignments indicated that nucleotide sequences were the more conservative and the most suitable taxonomic method [58,59,60]. In summary, whether the 13 amino acid sequences or nucleotides of the 13 PCGs was appropriate for phylogenetics was species dependent.

Our phylogenetic tree analysis indicated that all of our tested Ampullariidae species clustered on the same branch and all posterior probabilities were 100. These Pomacea species were clustered together and then with the genus *Marisa*. The species of the orders Architaenioglossa and Sorbeoconcha clustered together and then with Hypsogastropoda. This indicated that orders Architaenioglossa and Sorbeoconcha have a closer genetic relationship to the *Pomaceas*. Unexpectedly, *Ceraesignum maximum*, *Dendropoma gregarium*, *Eualetes tulipa* and *Thylacodes squamigerus* traditionally classified in the order Hypsogastropoda were isolated from this order and occupied the most external branch of the Bayesian tree.

### 2.8. Divergence Times of the Caenogastropoda

**Figure 9:**
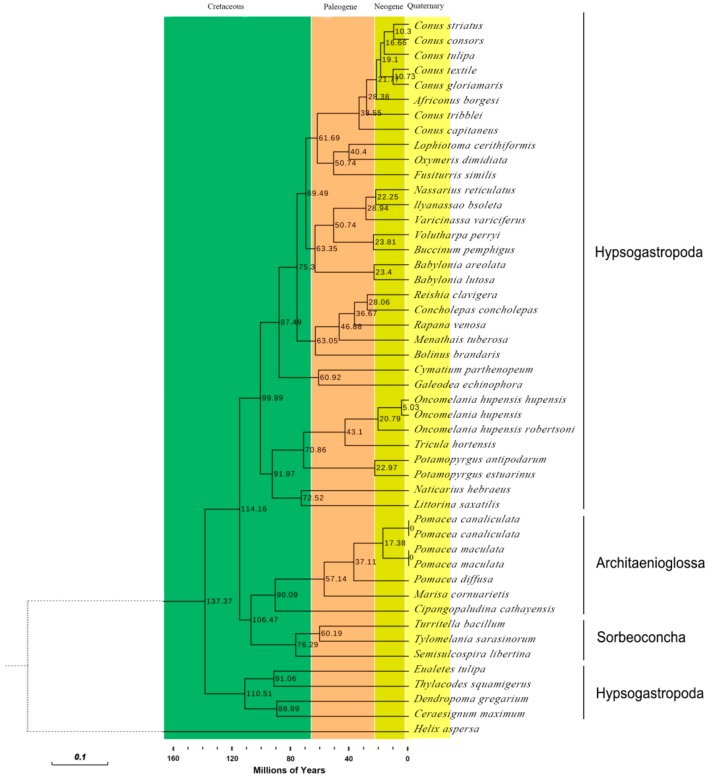
Chronogram for the 47 species of Caenogastropoda with a single out-group (*Helix aspersa*, NC_021747) based on the Bayesian topology calculated from analysis of the 13 PCGs. Divergence times were estimated using three calibrations. Numbers near the nodes indicated the average estimated divergence time estimated in Mya.

We estimated divergence times of the Caenogastropoda based on phylogenetic trees constructed using the Bayesian method and species differentiation time using the ML method [61]. The model MtMam + I + G + F was optimal with an AIC (Akaike Information Criterion) value of 621,746.50. The model JTT + I + G + F was suboptimal with an AIC value of 614,814.31, similar to the one for model MtMam + I + G + F (Figure 9). The JTT + I + G + F model was used for the molecular clock.

*Pomacea* taxonomy has been based on shell, egg mass and soft tissue morphology [5]. However, there are no clear criteria to distinguish between species because of intraspecific variation, most likely the result of environmental influences [62]. These unclear morphological criteria have confused the taxonomy and identification within the genus, especially between *P. canaliculata* and *P. maculate*. Most invasive apple snail populations have been identified as *P. canaliculata*, but many may belong to other species in a closely related complex. This difficulty has resulted in their informal naming as the “*P. canaliculata* group” [4]. Preliminary attempts at classifying these species using mitochondrial 12S, 16S, and COXI sequence have been successful [49]. In addition, *Pomacea* species in Japan have been classified using COXI sequence alignments [63]. However, these studies did not clearly distinguish between these organisms.

In our study, the divergence times indicated that *P. canaliculata* and *P. maculate* began to differentiate from other species about 17.38 million years ago (Mya) within the Neogene and then subsequently diverged with *P. diffusa 37.11* Mya within the Paleogene (Figure 9). This suggests that the longer the divergence time, the more likely differences in external morphology would be apparent. The divergence time of the orders Architaenioglossa and Sorbeoconcha was 106.47 Mya within the middle Cretaceous. The order Hypsogastropoda terminated their differentiation after three important differentiation nodes at 87.49, 91.97 and 99.99 Mya within the late Cretaceous, and converged with the Architaenioglossa-Sorbeoconcha cluster 114.16 Mya within the middle Cretaceous. Interestingly, the cluster gathered by *C. maximum*, *D. gregarium*, *E. tulipa* and *T. squamigerus* is traditionally classified in the order Hypsogastropoda and converged with the main Architaenioglossa–Sorbeoconcha-Hypsogastropoda cluster 137.37 Mya within the early Cretaceous.

Early phyletic evolution is primarily based on morphological characteristics. The development of modern molecular methods and techniques has provided additional information but also some new problems and challenges. These include how to comprehensively consider and balance position and function between morphology and modern molecular biology.

## 3. Materials and Methods

### 3.1. Sampling and Materials

Wild specimens of the three apple snails (*P. canaliculata*, *P. diffusa* and *P. maculate*) were collected in Ningxi Teaching and Research Farm of South China Agricultural University in Guangzhou (E 113°29′, N 23°5′) China on 2–25 March 2015. Voucher specimens were deposited in the Guangdong Engineering Research Center for Modern Eco-agriculture and Circular Agriculture, Guangzhou, China (Accession numbers: 004022015, 0050315 and 006012015). The dorsal muscle was preserved in 95% ethanol and stored at −70 °C until they were used for DNA extraction. Genomic DNA was isolated from muscle tissue as previously described [64]. Total DNA was eluted in sterile deionized water and was deposited at −20 °C. All animal experiments were conducted based on the guidelines and approval of the Animal Research and Ethics Committees of South China Agricultural University (AREC2003003, 15 March 2003).

### 3.2. Library Preparation for Sequencing

DNA sample quality and quantity were characterized by gel electrophoresis and UV spectroscopy using the Nano-Drop 2000 instrument (Thermo Scientific, Waltham, MA, USA). We isolated the high-quality genomic DNA to construct the DNA libraries containing insert sizes of 500 bp for paired-end sequencing. Paired-end reads of 100 bp were generated on an Illumina HiSeq2500 instrument (Illumina, Inc., San Diego, CA, USA) using sequencing protocols provided by the manufacturer.

### 3.3. Sequence Analysis and Annotation

Illumina paired-end sequencing reads were filtered on quality values and the low quality bases (quality < 20, *p*-error > 0.01) of 50 upstream and 30 downstream were trimmed. Using the Ampullariidae mitochondrial genomes from the NCBI database using bowtie2 (http://Bowtie-Bio.Sourceforge.Net/Bowtie2/Index.Shtml), the mitochondrial genome sequencing reads were captured. De novo assembly with paired-end sequencing reads was determined using SOAPdenovo2 (http://soap.genomics.org.cn/ soapdenovo.html). The protein-coding regions and ribosomal genes were identified using the Basic Local Alignment Search Tool (https://blast.ncbi.nlm.nih.gov/Blast.cgi) [65]. Species maps were drawn using OGDRAW (http://ogdraw.mpimp-golm.mpg.de/). Transfer RNA genes were predicted using a Cove score cutoff of 0.1 by tRNA scan-SE v.2.0 (http://lowelab.ucsc.edu/tRNAscan-SE/), combined with ARWEN software (http://mbio-serv2.mbioekol.lu.se/ARWEN/) [66,67] and then confirmed by MitoFish (http://mitofish.aori.u-tokyo.ac.jp/) to confirm the annotation. Strand asymmetry was calculated using the following formulas: AT skew = [A − T]/[A + T] and GC skew = [G − C]/[G + C] [68] to describe base composition. Repeat sequences were identified using Spectral Repeat Finder v1.1 [69]. Codon usage was determined for all PCGs. Long repeat analysis was used in the web-based REPuter (http://bibiserv.techfak.uni-bielefeld.de/reputer/) and included forward, reverse, and tandem repeats with minimal lengths of 30 bp and edit distances of <3 bp [70]. RSCU values [71] were obtained using MEGA 7 software (https://www.megasoftware.net/) [61]. Statistical analyses of the distributions and visualization of codon usage in the form of heat maps were conducted using R language with RSCU values, a measure of non-uniform usage of synonymous codons in a coding sequence [71]. The RSCU value was the number of times. A particular codon was observed relative to the number of times that the codon would be observed for a uniform synonymous codon usage i.e., all codons for a given amino acid exhibiting similar probabilities. The RSCU value in the absence of any codon usage bias was 1.00. A codon used less frequently than expected would have RSCU values <1.00, whereas codons used more frequently than expected would have RSCU values >1.00.

### 3.4. Phylogenetic Analyses

Phylogenetic analyses were conducted based on 47 mitochondrial genomes sequences of 47 species of Caenogastropoda (Table S1), using *Helix aspersa* (NC_021747) (Heterobranchia, Helicidae) as the out-group. All sequences were deposited in GenBank. We aligned amino acid sequences of PCGs in the 48 species using the MUSCLE program in MEGA 7. Our alignments of individual genes excluded the stop codon and the third codon.

We performed phylogenetic analysis by the Maximum Likelihood (ML) and Bayesian Inference (BI) methods. The best model MtMam + I + G + F based on the amino acid sequences in this study was selected using AIC (Akaike Information Criteria). Minimum and best values were fitted with ProtTest 3 (https://omictools.com/prottest-tool) with optimized parameters [72]. ML analysis was carried out using the RAxML 8.1.5 with 1000 bootstrap replicates based on the best-scoring protein substitution model determined automatically of the software [73]. Bayesian phylogenetic analysis with 10,000,000 generations was conducted with MrBayes 3.2.6 software (http://mrbayes.sourceforge.net/index.php) [74]. Four independent Markov chains were used at the same time with sampling every 100 generations. The BI Tree was reliable since the standard deviation of split frequencies was <0.01. The phylogenetic trees were generated using Evolview (http://www.evolgenius.info/evolview/) [75,76]. The NCBI taxonomy server (http://www.ncbi.nlm.nih.gov/taxonomy/) and DeepFin (http://deepfin.org/) were adopted for additional analyses.

The divergence times of the Caenogastropoda were estimated using MEGA 7.0 [77] with the RelTime-ML method and JTT + F + I + G modeling. Divergence times were presented in the Time Tree database (http://www.timetree.org/) [61]. Bayesian phylogenetic data were input to MEGA 7.0 to generate divergence times that ensured consistency between the phylogenetic tree and divergence times.

Sequence differences between three apple snails and other Sorbeoconcha and Architaenioglossa species were analyzed, and *P. canaliculata* (NC_024586) was used as the reference sequence. Sequences were aligned by BLAST and annular genetic similarity mapping was visualized using the CGView Comparison Tool (http://stothard.afns.ualberta.ca/downloads/CCT).

## 4. Conclusions

The gene characterization, arrangement and distribution of the three apple snails were canonically identical and consistent with the typical Mollusca except for the absence of *tRNA-Gln* in *P. diffusa*. The absence of an identifiable control region or d-loop was consistent with other Gastropoda. Bayesian phylogenetic analysis indicated that all Ampullariidae species including the apple snails clustered on the same branch with 100 posterior probabilities. The members of the genus *Pomacea* clustered together first and then with genus *Marisa*. The orders Architaenioglossa and Sorbeoconcha clustered together and then with the order Hypsogastropoda. Furthermore, the intergenic and interspecific taxonomic positions were explicit and clear. Unexpectedly, *C. maximum*, *D. gregarium*, *E. tulipa* and *T. squamigerus* traditionally classified in order Hypsogastropoda were isolated from it and located in the most external branch of the Bayesian inference topology tree. Divergence times of the Caenogastropoda indicated their evolutionary process covered four geological epochs: Quaternary, Neogene, Paleogene and Cretaceous. Early phyletic evolution is mainly based on morphological characteristics.

This study presents a characterization of mitochondrial genomes and insight into the phylogenetics of Caenogastropoda that may facilitate further investigation of species identification. This is essential for the implementation of effective management and control strategies.

## Figures and Tables

**Table 1:** Summary of gene/element feature of *P. canaliculata*, *P. diffusa* and *P. maculate*.

NO.	Gene/Element	Strand	Size (bp)	GC-Content (%)	Amino Acids (aa)	Inferred Initiation Codon	Inferred Termination Codon	One Letter Code	Anti-Codon	Intergenic Nucleotide*(bp)
1	*COXI*	H	1536	33.27–36.07	511	ATG	TAG/TAA			27–32
2	*COXII*	H	687	31.3–34.79	228	ATG	TAG/TAA			17–21
3	*tRNA-Asp*	H	68	14.71–17.65				D	GTC	0
4	*ATP8*	H	159	21.38–26.42	52	ATG	TAA			12–29
5	*ATP6*	H	699–714	26.90–29.41	232–237	ATG	TAG/TAA			15–28
6	*tRNA-Met*	L	64–66	27.69–34.38				M	CAT	23–35
7	*tRNA-Tyr*	L	65–68	31.34–41.18				Y	GTA	8–25
8	*tRNA-Cys*	L	62–65	21.54–29.03				C	GCA	5–19
9	*tRNA-Trp*	L	64–68	27.94–35.38				W	TCA	11–119
10	*tRNA-Gln*	L	62–64	34.38–35.48				Q	TTG	9–10
11	*tRNA-Gly*	L	66–67	19.40–20.9				G	TCC	24–31
12	*tRNA-Glu*	L	65–67	20.90–24.62				E	TTC	0
13	*rrns*	H	929–950	28.09–29.68						0–2
14	*tRNA-Val*	H/L	67–69	20.90–29.41				V	TAC	0–1
15	*rrnl*	H	1331–1345	25.47–27.14						0
16	*tRNA-Leu*	H	64–66	23.44–27.27				L	TAG	(−1)–0
17	*tRNA-Leu*	H	68–69	23.53–26.09				L	TAA	0
18	*ND1*	H	945–960	28.02–31.96	314–319	ATG	TAG/TAA			(−12)–9
19	*tRNA-Pro*	H	66–68	29.85–30.88				P	TGG	0
20	*ND6*	H	495	23.64–26.87	164	ATG	TAA			10–19
21	*Cytb*	H	1140	28.51–32.02	379	ATG	TAG/TAA			9–17
22	*tRNA-Ser*	H	66	33.33–34.85				S	TGA	20–30
23	*tRNA-Thr*	H/L	68–71	30.99–35.29				T	TGT	9–14
24	*ND4L*	H	297–312	26.92–27.95	98–103	ATA, ATG	TAG/TAA			(−7)–20
25	*ND4*	H	1341–1362	25.73–29.30	446–453	ATA, ATG	TAA			3–9
26	*tRNA-His*	H	64–65	16.92–21.88				H	GTG	0
27	*ND5*	H	1710–1728	28.13–31.42	569–575	ATG	TAA			(−20)–0
28	*tRNA-Phe*	H	68–69	30.88–33.33				F	GAA	141–1617
29	*COXIII*	H	780	33.72–37.69	259	ATA, ATG	TAA			47–67
30	*tRNA-Lys*	H	67–68	29.85–32.35				K	TTT	10–18
31	*tRNA-Ala*	H	68–71	23.19–28.17				A	TGC	29–46
32	*tRNA-Arg*	H	67–69	29.85–33.33				R	TCG	2–5
33	*tRNA-Asn*	H	69–71	21.13–24.64				N	GTT	19–30
34	*tRNA-Ile*	H	70–72	30.56–34.29				I	GAT	0–6
35	*ND3*	H	354	29.66–31.92	117	ATG	TAA			20–37
36	*tRNA-Ser*	H	68	30.88–32.35				S	GCT	0
37	*ND2*	H	1062–1071	27.12–30.91	353–356	ATG	TAG/TAA			4–6

Intergenic Nucleotide*(bp): positive values indicated the interval sequence of adjacent genes, and negative values indicated the overlapping of adjacent genes.

**Table 2:** Summary of the base composition of the mitogenomes for eight species of the orders Sorbeoconcha and Architaenioglossa in the Gastropoda.

Speics	*Pomacea canaliculata*	*Pomacea diffusa*	*Pomacea maculata*	*Marisa cornuarietis*	*Cipangopaludina cathayensis*	*Turritella bacillum*	*Tylomelania sarasinorum*	*Semisulcospira libertina*
Accession number	KU052865	KY008698	KY008699	NC_025334.1	NC_025577.1	NC_029717.1	NC_030263.1	NC_023364.1
Taxonomy	Caenogastropoda, Architaenioglossa	Caenogastropoda, Architaenioglossa	Caenogastropoda, Architaenioglossa	Caenogastropoda, Architaenioglossa	Caenogastropoda, Architaenioglossa	Caenogastropoda, Sorbeoconcha	Caenogastropoda, Sorbeoconcha	Caenogastropoda, Sorbeoconcha
Length (bp)	16,965	16,640	15,516	15,923	17,157	15,868	16,632	15,432
A (%)	32.61	30.13	30.81	28.94	26.74	28.85	29.49	31.4
T (%)	39.85	39.62	41.13	41.04	44.51	35.88	35.65	34.76
C (%)	12.03	14.24	12.81	13.34	8.28	16.21	16.56	17.78
G (%)	15.51	16.02	15.25	16.68	20.48	19.06	18.3	16.06
AT (%)	72.46	69.75	71.94	69.98	71.25	64.73	65.14	66.16
AT-skew	−0.0998	−0.1361	−0.1433	−0.1729	−0.2493	−0.109	−0.0946	−0.0508
GC-skew	0.1263	0.059	0.0868	0.1113	0.4243	0.0809	0.0499	−0.0507
Length (aa)	3724	3737	3733	3712	3727	3737	3755	3750
AT (%) (all)	70.71	67.98	71	68.98	70.29	63.89	64.29	65.66
AT (%) (3rd)	72.38	76.02	85.61	82.34	79.38	68.75	68.58	69.35
AT-skew	−0.2099	−0.2064	−0.2022	−0.2312	−0.3243	−0.119	−0.1033	−0.0642
GC-skew	0.0844	0.0466	0.0697	0.0764	0.3843	0.0862	0.0696	−0.0397
Length (bp)	1334	1345	1331	1359	1387	1357	1382	1347
AT (%)	73.54	72.86	74.53	72.77	72.17	68.53	66.93	68.08
AT-skew	0.0051	0.0163	0.006	−0.0253	−0.1109	−0.073	−0.107	−0.06
GC-skew	0.1501	0.1397	0.1622	0.2324	0.4456	−0.002	−0.1116	−0.0791
Length (bp)	929	950	934	977	895	949	899	951
AT (%)	71.91	70.32	70.99	69.81	66.03	67.76	64.29	66.56
AT-skew	0.012	0.021	0.0377	−0.0264	−0.0592	−0.154	−0.1142	−0.0142
GC-skew	0.249	0.1773	0.1956	0.2542	0.4474	−0.033	−0.1153	−0.1509
Length (bp)	1473	1484	1479	1500	1471	1486	1482	1489
AT (%)	71.89	71.56	72.08	68.07	72.33	64.8	64.3	65.14
AT-skew	−0.0293	−0.0226	−0.0263	−0.0245	−0.0602	−0.09	−0.0766	−0.0124
GC-skew	0.0821	0.0616	0.0847	0.0814	0.2776	0.0554	0.0057	−0.0405

## Supplementary Materials

Supplementary materials can be found at http://www.mdpi.com/1422-0067/19/11/3646/s1.
